# Spatial modes for transmission of chikungunya virus during a large chikungunya outbreak in Italy: a modeling analysis

**DOI:** 10.1186/s12916-020-01674-y

**Published:** 2020-08-07

**Authors:** Giorgio Guzzetta, Francesco Vairo, Alessia Mammone, Simone Lanini, Piero Poletti, Mattia Manica, Roberto Rosa, Beniamino Caputo, Angelo Solimini, Alessandra Della Torre, Paola Scognamiglio, Alimuddin Zumla, Giuseppe Ippolito, Stefano Merler

**Affiliations:** 1 Center for Information Technology, Fondazione Bruno Kessler, Trento, Italy; 2 National Institute for Infectious Diseases “Lazzaro Spallanzani” IRCCS, Rome, Italy; 3 Centro Agricoltura Alimenti e Ambiente, Università di Trento, San Michele all’Adige, TN Italy; 4 Dipartimento di Biodiversità ed Ecologia Molecolare/Centro Ricerca e Innovazione, Fondazione Edmund Mach, San Michele all’Adige, Italy; 5 Dipartimento di Sanitá Pubblica e Malattie Infettive, Sapienza University of Rome, Rome, Italy; 6 Division of Infection and Immunity, Center for Clinical Microbiology, University College London, London, UK; 7 the National Institute of Health Research Biomedical Research Centre at UCL Hospitals, London, UK

**Keywords:** Chikungunya, Transmission chain, Spatiotemporal spread, Transmission distance

## Abstract

**Background:**

The spatial spread of many mosquito-borne diseases occurs by focal spread at the scale of a few hundred meters and over longer distances due to human mobility. The relative contributions of different spatial scales for transmission of chikungunya virus require definition to improve outbreak vector control recommendations.

**Methods:**

We analyzed data from a large chikungunya outbreak mediated by the mosquito *Aedes albopictus* in the Lazio region, Italy, consisting of 414 reported human cases between June and November 2017. Using dates of symptom onset, geographic coordinates of residence, and information from epidemiological questionnaires, we reconstructed transmission chains related to that outbreak.

**Results:**

Focal spread (within 1 km) accounted for 54.9% of all cases, 15.8% were transmitted at a local scale (1–15 km) and the remaining 29.3% were exported from the main areas of chikungunya circulation in Lazio to longer distances such as Rome and other geographical areas. Seventy percent of focal infections (corresponding to 38% of the total 414 cases) were transmitted within a distance of 200 m (the buffer distance adopted by the national guidelines for insecticide spraying). Two main epidemic clusters were identified, with a radius expanding at a rate of 300–600 m per month. The majority of exported cases resulted in either sporadic or no further transmission in the region.

**Conclusions:**

Evidence suggest that human mobility contributes to seeding a relevant number of secondary cases and new foci of transmission over several kilometers. Reactive vector control based on current guidelines might allow a significant number of secondary clusters in untreated areas, especially if the outbreak is not detected early. Existing policies and guidelines for control during outbreaks should recommend the prioritization of preventive measures in neighboring territories with known mobility flows to the main areas of transmission.

## Background

Understanding and quantifying the spread of mosquito-borne infections over space and time is critical for assessing transmission risks, strengthening entomological and human surveillance, and guiding the implementation of vector control measures, blood transfusion safety, vaccine delivery, and health system strengthening. Several recent studies based on advanced statistical modeling techniques have elucidated the spatiotemporal spread of arboviruses transmitted by *Aedes aegypti* in both endemic [1–3] and non-endemic areas [4, 5]. All these show a dominant focal nature [6, 7] for the spatial transmission despite geographic and socio-economic differences in affected populations. The majority of cases are distributed over short distances (< 1 km), compatible with the short-ranged dispersal of mosquitoes [8] and human walking distances, resulting in focal areas of intense transmission. Occasionally, long-distance travel of arbovirus-infected individuals results in the seeding of further foci of infection at more distant areas where the relevant mosquito vectors are prevalent. The relative contributions of different spatial transmission scales during outbreaks remain unclear and require definition. Furthermore, there are no data on the dynamics of arbovirus dispersal in areas where the main mosquito vector is *Aedes albopictus*, a more exophilic species compared to *A. aegypti* and characterized by longer flight ranges [9, 10]. The widespread and abundant presence of *A. albopictus* in temperate, non-endemic areas of Europe, the USA, northern China, the Korean Peninsula, and southern Australia [11] has created conditions for transmission of arboviral tropical diseases such as dengue [12], chikungunya [13, 14], and potentially Zika [15] and yellow fever [16]. The expected expansion of its habitat [17] in the future will put an even larger territory at risk of sustained arboviral transmission. A large chikungunya outbreak in Italy transmitted by *A. albopictus* started in early summer 2017 in the coastal holiday town of Anzio and spilled over to multiple sites within the Lazio region [18], Italy. The outbreak consisted of 414 reported human cases until the end of the outbreak in November 2017. We report transmission chains and the relative weight of transmission at different geographic scales and quantify the spatial and epidemiological dynamics of the outbreak.

## Methods

### Geographical setting and study population

In 2017, the second outbreak ever of chikungunya in continental Europe occurred in the Lazio region of Italy leading to a total of 414 cases (200 confirmed, 202 probable, 12 under investigation) within the region [18]. The Lazio region has 6 million inhabitants, almost half of which live in the metropolitan area of Rome. According to available estimates, each year, a median of 14 chikungunya and 98 dengue cases are imported in the region from endemic countries via the Rome Fiumicino Airport (the main international airport in Italy) [19]. The main area of transmission was in the town of Anzio, 62 km south of Rome, and involved 317 persons with a direct epidemiological link. The town is a popular seaside destination for many inhabitants of Rome and neighboring cities, who commute to the coast on a daily basis or spend the summer in their own beach houses, resulting in a significant outflow and inflow of people during warmer months. Although the outbreak was first detected on September 7, 2017, the first recorded symptom onset was retrospectively estimated to have occurred at the end of June [18]. After the identification of autochthonous chikungunya transmission, insecticide spraying was carried out in most involved areas. Despite intensive interventions, transmission continued for about 2 months, with the last symptom onset date recorded in Anzio on November 5, 2017 [18], suggesting that declining temperatures may have played a role in bringing transmission under control [20, 21].

### Data collection

Data on chikungunya notifications were collected by the Regional Service for Surveillance and Control of Infectious Diseases – Lazio Region (SERESMI) [18] according to the framework of the regional integrated surveillance system for chikungunya, dengue, and Zika based on the national plan for surveillance and control of arboviruses transmitted by *Aedes* mosquitoes [19]. Data included the geographic location of the residence and the date of symptom onset (range, June 26 to November 5, 2017) for 412 of the 414 cases (two cases with missing information were excluded from the analysis). In addition, epidemiological investigations were performed, consisting in the administration of a questionnaire aimed at ascertaining the patients’ history of travel during the 15 days before the symptom onset. A full description of the data collection methodology can be found in [18].

### Transmission chain reconstruction model

We apply a previously developed model [5] which is able to derive a probabilistic inference of transmission chains from the spatiotemporal structure of data; more specifically, the model defines the likelihood that each case was infected by any of the other cases, based on a definition of the force of infection that depends on the time of symptom onset of potential infectors and on the spatial coordinates of their residence. By applying this model to data from the Anzio outbreak, we determined three main scales of transmission: (i) “focal” transmission involving individuals residing in spatiotemporal proximity to the residence of an infector, (ii) “local” transmission of infections occurring among residents from the same area that were not considered focal, and (iii) “exported cases” defined as not residing in but with a known epidemiological link to Anzio. The model assumes a gamma-distributed generation time (i.e., the time elapsed between the infection of a primary case and of its secondary cases) and that the probability of focal transmission declines exponentially with the distance from an infector. Using a probabilistic approach, the model is able to discriminate local from focal transmission and to identify the most likely source of infection for focal cases. We did not infer the most likely infector for exported cases because their location in Anzio at the time of infection was unknown; instead, we considered them as transmissions within Anzio, and we kept them in the dataset as potential sources of further focal transmissions in the municipality where they reside. Eventually, the transmission chain was defined by the list of the most likely infectors for each case (who infected whom); accordingly, the transmission distance was defined as the distance between the residences of a secondary case and of its associated infector. The model provides estimates of parameters related to the spatial scale of focal transmission and of the generation time (i.e., the time between successive infections in a transmission chain). Parameter estimation was based on Markov Chain Monte Carlo. Here, we present the results relative to a single transmission chain, corresponding to the one with the highest likelihood. In Additional File 1: Sections 1.2 and 1.4, we report full details on the model and calibration procedure, as well as a sensitivity of results with respect to alternative stochastic reconstructions of the transmission chain and to several variations on model specifications.

### Focal clusters

We defined a focal cluster as the set of secondary cases belonging to a unique chain of focal transmission, directly and indirectly originated by a single index case (i.e., an exported or locally transmitted case). For each cluster, we defined its size as the total number of secondary cases involved, and its radius as the maximum distance between any case in the cluster and the index case.

### Virtual outbreaks

To validate the results on the estimated transmission distances, we simulated virtual outbreaks in the Anzio area by using a branching process model informed with probability distributions estimated via the transmission chain reconstruction model. Starting from a single index case, a number of reported secondary cases were sampled for each patient from the estimated distribution, varying over time; the time of symptom onset and location of residence of secondary cases were sampled from the estimated distribution of the generation time and of transmission distances. The model also accounts for the presence of asymptomatic cases and of underreporting [22] (see Additional File 1: Section 1.5 for further details).

### Alternative intervention scenarios

To estimate the impact of alternative intervention scenarios, we performed additional simulations with a spatial mechanistic model accounting for the presence over time and space of infected mosquitoes and for the effect of both human mobility in spreading the infection and insecticide spraying in containing it. We assumed the interventions to be performed within a given radius from the residence of a notified case and after a given time delay from his/her symptom onset date. Insecticides are estimated to kill about 86% of existing mosquitoes in the sprayed area, and the mosquito population is assumed to recover to pre-intervention abundance after 2 weeks [23]. We tested the effectiveness of intervention protocols with different values of the radius of spraying, the intervention delay, and the date of outbreak detection; baseline values for these three parameters were 200 m, 6 days, and September 7, respectively, to reproduce the actually implemented interventions. In addition, we considered as a sensitivity analysis the effectiveness of interventions when infections are restricted to occur only within 1 km or within 200 m. Full details are reported in Additional File 1: Section 1.6.

## Results

**Fig. 1:**
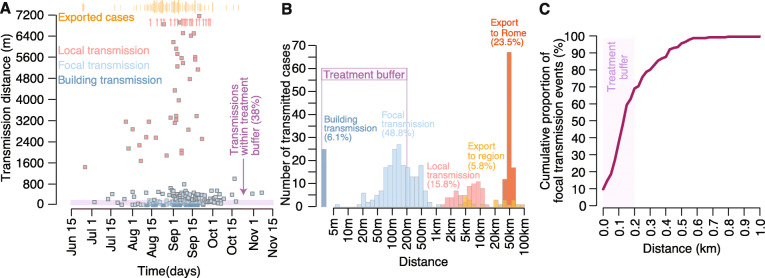
Estimated transmission distances by scale of transmission. **a** Individual chikungunya cases are shown by date of symptom onset and estimated distance from its infector; transmission within the same building (i.e., at distance 0) is a special case of focal transmission and is displayed with a darker blue compared to other focal transmissions; red arrows at the top of the graph indicate cases transmitted locally over distances larger than the maximum scale of the graph; the timing of exported cases is indicated with vertical orange bars above the graph, and the size of the bar is proportional to the number of cases occurring on that day; the pink horizontal band at the bottom of the graph defines the radius of the buffer (200 m) surrounding a notified case within which insecticide treatments are conducted. **b** Distribution of the number of cases by transmission distance for different spatial scales; the *x*-axis is log-scaled. **c** Cumulative distribution of transmission distances for the focal component only; the 200-m treatment buffer is displayed

A total of 412 chikungunya cases were included in the transmission chain reconstruction model. The results showed that 54.9% of all transmission events were due to focal spread (including 6.1% of cases occurring in individuals living in the same building as the infector), 15.8% occurred locally within a range comprised between 1.5 and 12.9 km, and the remaining 29.3% were exported cases, largely over distances beyond 20 km (Fig. 1 and Table 1).

**Table 1:** Model classification of chikungunya cases by scale of transmission and residence

	Scale of transmission				
	Numbers	Focal		Local (%)	Exported (%)
		Within building (%)	Outside building (%)		
Residence					
Anzio	190	14 (7.4)	151 (79.5)	25 (13.1)	0 (0.0)
Rome	169	10 (5.9)	34 (20.1)	28 (16.6)	97 (57.4)
Others	53	1 (1.9)	16 (30.2)	12 (22.6)	24 (45.3)
Total	412	25 (6.1)	201 (48.8)	65 (15.8)	121 (29.3)

During the central part of the outbreak (from mid-August to the end of September 2017), about 16 cases/week were exported from the municipality of Anzio to other areas of the region, 8 cases/week were transmitted locally, and 29 cases/week were transmitted focally. The median distance of focal transmission was 127 m (Fig. 1c), and 69% of focal infections occurred within 200 m, i.e., the distance recommended by the national guidelines to perform insecticide spraying aimed to interrupt or reduce spreading of the outbreak. When considering all cases, including locally transmitted and exported cases, the proportion of transmission events covered by the treatment buffer reduces to 38% (Fig. 1a, b). The model estimated an average generation time of 12.4 days (95%CI of the mean, 11.7–13.3). The individual variability of the generation time, computed as the 95% quantiles of the estimated probability distribution, ranged between 5.6 and 22.1 days.

**Fig. 2:**
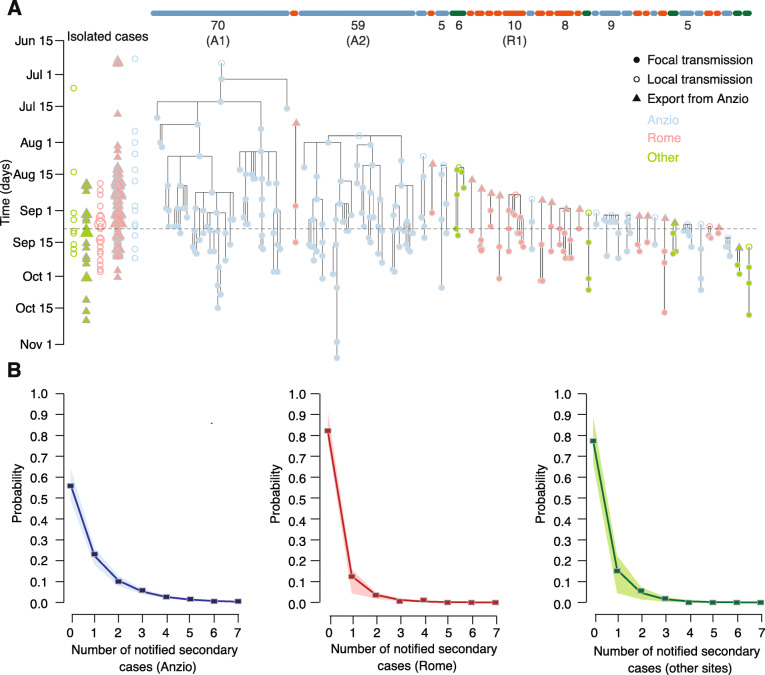
Reconstructed transmission chains. A maximum likelihood transmission chains in the 2017 chikungunya outbreak in Lazio; the vertical dimension represents the time of symptom onset of individual cases. Horizontal bars at the top of the graph denote distinct focal clusters and are colored by the location of the cluster (blue, Anzio; red, Rome; green, others). Below the bars, we reported the total cluster size (for sizes ≥ 5) and the corresponding label (for sizes ≥ 10: A1 and A2, occurring in Anzio, and R1, occurring in Rome). Clusters are ordered horizontally by the date of the corresponding index case. Shown on the left-hand side of the panel are cases with no secondary focal transmission and no identified infector (“isolated cases”), disaggregated by whether they were exported or locally transmitted cases and color-coded by location; the size of the points is proportional to the number of isolated cases occurring in a given day, location, and scale of transmission (the minimum size corresponds to a single isolated case). **b**. Distributions of the number of secondary cases transmitted focally by each case for different locations of infectors; points, crude estimates from the transmission chains; lines and shaded areas, mean and 95%CI from a negative binomial fit of the points

In Anzio, the reconstruction of transmission chains identified two main clusters of sustained focal transmission (denoted with A1 and A2, Fig. 2a), comprising 70 and 59 secondary cases, respectively, and representing over two thirds of all cases notified in the municipality. We identified a likely infector for 87% of all cases (165/190); of the remaining twenty-five locally transmitted cases (13%), two originated from the main clusters, ten gave rise to small focal clusters with an average size of 3.6, and 13 remained isolated cases not resulting in focal transmission. Outside of Anzio, focal transmission was rare (80% of cases were isolated, Fig. 2b), and when it occurred, it was limited to small clusters of average size 2.9; the largest cluster occurred in Rome and involved 10 secondary cases (indicated as R1 in Fig. 2a).

**Fig. 3:**
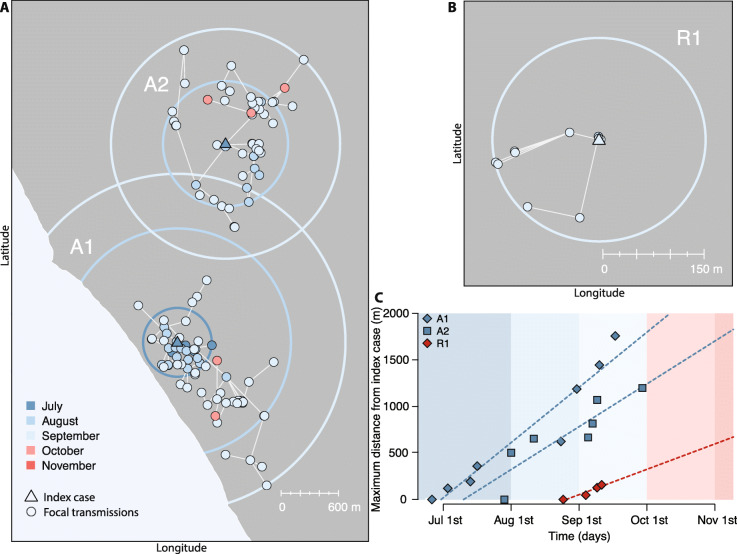
Spatiotemporal spread of focal clusters. **a** Main clusters of focal transmission in Anzio (A1 and A2); colors indicate the date of symptom onset; circles are centered on the index case and have the same radius as the maximal distance of cases from the index case (cluster radius) at the end of the corresponding month. **b** The main cluster of focal transmission in Rome (R1). **c** Expansion of the radius over time for clusters A1, A2, and R1. Points are shown at each new record for the cluster radius. Dashed lines represent linear regressions across these points, and their slope indicates the rate of cluster expansion

The spatial spread over time of the three largest clusters (A1, A2, and R1) is represented in Fig. 3c. Cluster A2 started at the end of July, when cluster A1 had been active for 1 month, cumulating six secondary cases within a radius of 360 m. The index case of cluster A2 was located at least 2.1 km apart from any case that occurred until then in A1, i.e., much further than the largest transmission distance for cases classified by the model as focal (~ 1 km). Cluster R1 started in Rome at the end of August by an infected case with no connection to Anzio who likely acquired infection locally from one of the ongoing transmission clusters in the city. All three clusters expanded geographically over time by increasing their radius in an approximately linear way and at a rate comprised between 300 and 600 m per month (Fig. 3c).

**Fig. 4:**
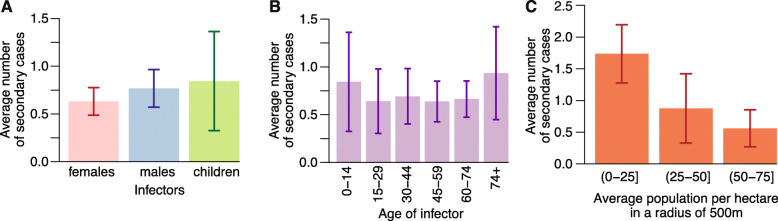
Determinants of transmission. **a** Average number of secondary cases and 95% credible intervals by demographics of the infector. **b** As in **a**, but by age of the infector. **c** As in **a**, but by average human density around the residence of infector

The model did not identify significant heterogeneities in the number of secondary infections by demographic groups (males, females, or children below 16 years of age) or by age group (Fig. 4). However, individuals who lived in less densely populated areas produced a significantly larger number of secondary cases (Fig. 4c; see Additional File 1: Section 5.1 for statistical details).

**Fig. 5:**
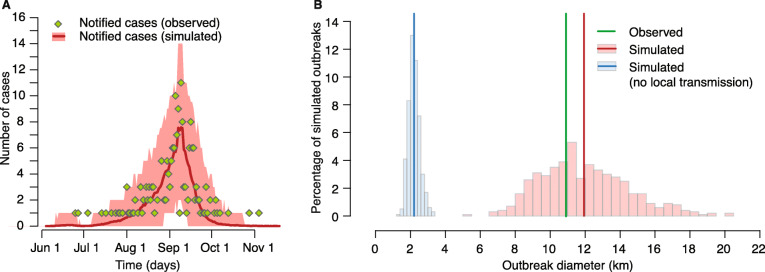
Comparison between observed and simulated outbreaks in Anzio using a branching process informed with estimates from the transmission chain reconstruction model. **a** Temporal evolution of 1000 simulated outbreaks (solid lines, average; shaded areas, 95%CI), compared with observed daily chikungunya notifications (*N* = 190). **b** Histograms of final diameter (maximum distance between any two cases) in simulated outbreaks by considering local transmission (pink) or without considering local transmission (light blue); vertical lines represent the observed final outbreak diameter in Anzio (green) and the averages of the simulated distributions (blue and red)

Virtual outbreaks simulated by the branching process well reproduced the spatial and temporal behavior of transmission within the Anzio area. The epidemic curve and the final diameter (defined as the maximum distance between any two cases) of the observed outbreak were closely reproduced by simulated outbreaks (Fig. 5). In particular, the average simulated outbreak diameter was 11.9 km (95%CI, 7.8–17.4), against an observed value of 10.9 km. Notably, if local transmission was not allowed in the simulated outbreaks, the diameter decreased to about one fifth of the observed value (average, 2.2 km; 95%CI, 1.7–3.0, Fig. 5b), showing the importance of local transmission for epidemic dispersal. This result did not change with different assumptions on underreporting made in the branching process (see Additional File 1: Section 3).

**Fig. 6:**
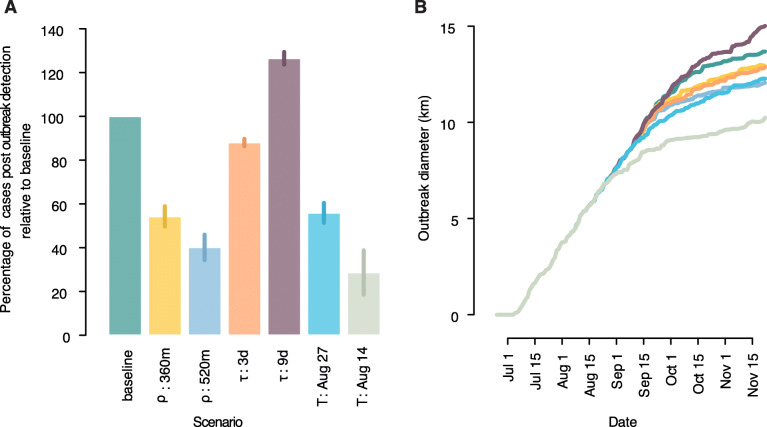
Impact of alternative intervention protocols. **a** Proportion of the number of cases observed after outbreak detection for different intervention protocols with respect to the baseline. *ρ*, the radius of insecticide spraying (baseline, 200 m); *τ*, average delay of intervention with respect to symptom onset (baseline, 6 days). *T*, date of outbreak detection (baseline, September 7). **b** Outbreak diameter over time by intervention protocol. Colors as in **a**

Simulations on alternative intervention protocols showed that expanding the radius of insecticide spraying from 200 to 360 m would be able to almost halve (− 46%) the number of cases observed after outbreak detection; a further increase of the radius to 520 m would result in a slightly larger reduction of 60% (Fig. 6). Keeping the radius at 200 m while halving the average delay between symptom onset and intervention (from 6 to 3 days) would allow a reduction of 12%, while a slow-down of the average response (from 6 to 9 days) would result in 26% excess cases. Finally, anticipating the date of outbreak detection by approximately one or two generations of transmission would reduce the number of cases observed after detection by 44% and 71%, respectively. Figure 6 also shows the effect of treatment on the rate of cluster expansion and on the final cluster size. Current reactive intervention protocols would be much more effective in outbreaks where local transmissions are not common (47% less cases, see Additional File 1: Section 4) or where transmission largely occurs within 200 m, such as in rural villages with limited human mobility [2] (81% less cases).

## Discussion

This is the first study quantifying the relative contributions of focal transmission and longer-distance disease spread mediated by human mobility in a large arboviral outbreak in Europe. Our data revealed that focal transmission accounted for only about 55% of new infections, and only 38% of all cases were transmitted to residents living within a distance of 200 m from the index case. About 45% of infections were transmitted at distances between 2 and 100 km. These are also the first quantitative results describing the spatiotemporal spread of an outbreak transmitted by *A. albopictus* and in Europe.

Our results are important for policymakers for setting public health priorities and guidelines for outbreak investigation and measures for vector control. National guidelines for outbreak control and prevention of spread based on WHO recommendations were adopted and implemented during the Lazio outbreak. The buffer distance adopted for insecticide spraying to eliminate mosquitoes was 200 m. Our study estimates that 62% of cases were transmitted to residents living at distances larger than 200 m; therefore, the majority of cases that are transmitted before interventions (or despite them, given that a proportion of mosquitoes survives insecticide treatment [23]) will have a chance to ignite further spread in untreated areas, making control of an outbreak significantly more difficult. Indeed, human mobility can contribute to seed a relevant number of new foci of transmission over several kilometers. During the Lazio outbreak, a significant number of cases (15%) were transmitted locally over distances of 1.5–15 km, i.e., at distances compatible with the usage of transportation means, and 30% of cases were exported to locations at more than 20 km from the core area of transmission. We have shown that the effect of transportation-mediated human movement at least halves the effectiveness of the insecticide protocol compared to a theoretical scenario where only focal transmission is allowed (Appendix File 1: Section 4). Furthermore, the outbreak in Lazio was detected more than 2 months after the beginning of the outbreak, corresponding to at least 5 generations of transmission. These results support previous research suggesting the need for improving preparedness towards earlier detection and response [24, 25], which can be achieved by increasing the awareness of physicians and health personnel with respect to the transmission risks and clinical manifestations of arboviral diseases [18, 26]. Although the specificity of Anzio as a summer holiday destination and its proximity to a large metropolitan city likely inflated the probability of local transmission and exportation (for example compared to the 2007 outbreak in Emilia Romagna, Italy [27, 28]), it is not unlikely to observe similar patterns in future outbreaks: many touristic destinations in temperate areas are towns with low human population density and great suitability for mosquito populations and therefore with a high risk of arboviral transmission. The larger-than-expected role of human mobility and the partial effectiveness of reactive interventions [29] call for the implementation of preventive vector control activities targeting all municipalities at risk of autochthonous transmission that are adjacent to affected areas. In Europe, larvicidal strategies focused on public spaces have been shown to be cost-effective in preventing arbovirus transmission in highly structured urban environments in Europe, even in areas with moderate to low mosquito infestation [30].

As for focal transmission, accounting for about 55% of all cases, we estimated an average transmission distance of about 130 m. Despite important behavioral differences between *A. albopictus* and *A. aegypti* in spatial dispersal and resting preference [8–10], our estimate is in remarkable agreement with several other results obtained for infections transmitted by *A. aegypti* using different methods and in highly diverse settings (e.g., geographic area, circulating virus, level of endemicity) [1–7]. In addition, the expansion of focal clusters in Lazio proceeded at a rate between 300 and 600 m per month, which is also remarkably in line with analogous estimates for dengue in Brazil [5]. Thus, the dynamics of focal spread seem quite independent of the *Aedes* vector species, as well as of the specific disease and geographical or epidemiological setting, suggesting that they are rather driven by the limited variability of human walking distances.

Few long-distance transmissions (about 13%) resulted in the ignition of new infectious foci in Lazio outside of Anzio, mainly in Rome, and the size of the resulting clusters was always small (average 2.6 cases). This finding is in line with results from theoretical mathematical models that show that vector-borne transmission might be lower in areas with high human population density [21].

Identifying transmission chains for infections is a difficult task and prone to mistakes. Possible sources of bias in this type of analysis may come from missed cases due to asymptomatic infections and underreporting. While asymptomatic rates are low for chikungunya (about 18% [22]), underreporting may have been an issue in the earlier part of the outbreak. Indeed, because the transmission was detected over 2 months after the beginning of the outbreak, most of the earlier cases were discovered retrospectively and the outbreak’s case zero could not be identified [18]. Spatial heterogeneity in underreporting is also possible, because retrospective surveillance might have focused preferentially on areas with known chikungunya clusters, missing some other areas of focal transmission. In general, previous works have demonstrated that the transmission chain reconstruction model is robust with respect to undernotification: spatiotemporal parameters of infection were systematically inferred from synthetic data even when 50% or more of cases were removed [5]. Underreporting rates larger than 50% are unlikely for chikungunya: even during the 2007 outbreak in Emilia Romagna, Italy, an overall underreporting rate of 22% for symptomatic cases was estimated via serological studies [22]. Furthermore, our branching process validated our estimates of the transmission distance distribution, being able to correctly reproduce the spatiotemporal spread of the outbreak in Anzio independently of assumptions on the reporting rate. Finally, our qualitative and quantitative conclusions were remarkably robust with respect to a number of alternative model formulations and to stochastic variability in the inferred transmission chains (see Additional File 1: Section 2).

Our analysis implicitly assumes that infected mosquitoes transmitting the infection to other individuals live in the close vicinities of a case’s residence. This assumption is also at the basis of infection control protocols via insecticide spraying in a radius of the case’s residence and derives from the highly focal nature of mosquito-borne infections, demonstrated by several studies [1–7]. However, mosquitoes may also acquire the virus by biting infectious individuals in sites different from the infector’s residence, such as parks, gardens, and other public spaces: for example, using contact tracing data from a dengue outbreak in Australia, Vazquez-Prokopec and colleagues [4] linked at least 10% and up to 57% of human cases to exposures in out-of-home locations. We expect out-of-home exposure for chikungunya to play a less important role compared to dengue, given that chikungunya has higher symptomatic rates (therefore, infected individuals are more likely to stay at home) and that chikungunya transmission by asymptomatic individuals is uncertain. Nonetheless, further research on the importance of out-of-home exposures would help to better elucidate the contribution of public places in arboviral transmission, providing additional useful indications for control.

## Conclusions

During outbreaks of arboviral infections, a large proportion of secondary cases are transmitted to individuals who live much further than 200 m from the residence of a case, i.e., the currently adopted radius for intervention control. In this way, new foci of transmission can be seeded in untreated areas over several kilometers, especially if the outbreak has gone initially undetected. To improve outbreak control, policies should recommend the prioritization of preventive measures in neighboring territories with known mobility flows to the main areas of transmission.

## Supplementary information

**Additional file 1: Appendix.**

## Data Availability

The datasets used and/or analyzed during the current study are available, only for sections non-infringing personal information, from the corresponding author on reasonable request and stored at INMI’s Library.
