# Supplementary material for: Spatial modes for transmission of chikungunya virus during a large chikungunya outbreak in Italy: a modeling analysis
Source: BMC Med. 2020 Aug 7;18:226. doi: 10.1186/s12916-020-01674-y (PMC7412829; doi:10.1186/s12916-020-01674-y)
Supplement: Supplementary file 1 — Additional file 1: Appendix. [file 12916_2020_1674_MOESM1_ESM.pdf]

Appendix

Focal transmission and the role of human mobility in a large chikungunya outbreak in Italy: a modeling analysis

Authors:
Giorgio Guzzetta[1*], Francesco Vairo[2*$,] Alessia Mammone[2], Simone Lanini[2], Piero Poletti[1], Mattia Manica[3], Roberto Rosa[`3], Beniamino Caputo[4], Angelo Solimini[4], Alessandra Dellatorre[4], Paola Scognamiglio[2], Alimuddin Zumla[5], Giuseppe Ippolito[6#], Stefano Merler[1#]

Institutional Affiliations:

1. Center for Information Technology, Fondazione Bruno Kessler, Trento, Italy
2. Regional Service for Surveillance and Control of Infectious Diseases (SERESMI), National Institute for Infectious Diseases "Lazzaro Spallanzani" IRCCS, Rome, Italy

3. Dipartimento di Biodiversita' e Ecologia Molecolare/Centro Ricerca e Innovazione, Fondazione Edmund Mach, San Michele all'Adige, Italy

4. Dipartimento di Sanita' Pubblica e Malattie Infettive, Sapienza University of Rome

5. Division of Infection and Immunity, Center for Clinical Microbiology, University College London; and the National Institute of Health Research Biomedical Research Centre at UCL Hospitals, London, UK.

6.National Institute for Infectious Diseases Lazzaro Spallanzani-IRCCS, Rome, Italy

[*] Joint first authors
[#] Joint senior authors
[$] Corresponding author

1. Details on methods

1.1. Additional information on data

Human population density data at a resolution of 250m were obtained from the Global Human Settlement database [1] and administrative unit shapefiles were downloaded from the National Institute of Statistics [2]. The categorical location of cases was assigned on the basis of the place of residence with respect to the municipality shapefiles for Anzio and Rome; cases whose residence was outside the borders of either municipality were classified as "other". Table S1 summarizes the information from epidemiological questionnaires administered during the outbreak. These data were used for the definition of cases exported from Anzio, i.e. those with an epidemiological link to this town in the days before symptom onset. Exported cases were excluded from the transmission chain reconstruction in the main analysis.

Table S1. *Data from epidemiological questionnaires (including all cases for which the geographical location is known).*

			epidemiological link	
		total	export from Anzio	transmission within same area
residence	Anzio	190	-	190
	Rome	169	97	72
	other	63	24	29
	total	412	121	291

1.2. Baseline model (main analysis)

The model assumes the following expression for the force of infection to which any case *j* was exposed at time *t*:

$$\lambda_j(t) = \sum_{i \in N_j(t)} \left(\alpha + \beta K\left(d_{ji}; \eta\right)\right) \Gamma(t - E_i; a, b)$$

where $N_j(t)$ is the set of notified chikungunya cases resident in the same location of *j* who have been infected before time *t*. α and β are two scaling factors for the transmission rates relative to the local and focal mode of infection, respectively; $K\left(d_{ji};\ \eta\right)$ is a spatial kernel with parameter η, regulating the distance-dependent probability of focal transmission; d_{ji} is the distance between the residences of individuals *j* and *i*; E_i is the time at which individual *i* acquired infection; and $\Gamma(t;\ a, b)$ is the gamma-distributed (with shape a and rate b) probability distribution function of the generation time, i.e. the time elapsed between infection times in a primary host and in its secondary cases. Since only information on the dates of symptom onset are available, the set of infection times $\boldsymbol{E} = \{E_j\}$ was unknown and was considered as a set of nuisance parameters (augmented data) to be estimated together with model parameters. The generation time is assumed to be to be gamma-distributed with shape *a* and rate *b*; it reflects a time-varying level of infectiousness of cases after their initial infection and accounts for the length of incubation periods in both humans and mosquitoes (intrinsic and extrinsic incubation periods), the duration of human infectiousness and the lifespan of mosquitoes. The spatial kernel accounts for focal transmission and is assumed to follow a negative exponential distribution with parameter η, $K\left(d_{ji};\ \eta\right) = \frac{1}{\eta} e^{-\eta d_{ji}}$ [3].

For each j, we define a most likely infector, k_j, such that

$$\beta K(d_{k_j j};\ \eta)\Gamma(E_{k_j} - E_i; a, b) \ = \ max_i\ (\beta K(d_{ij};\ \eta)\Gamma(E_j - E_i; a, b));$$

If the focal component of the most likely infector, $\beta K(d_{k_j j};\ \eta)$, is lower than the local component α, the case is classified as locally transmitted and no specific infector is identified; otherwise k_j is considered as the source of infection for j. The likelihood that j acquired infection at time E_j was defined as

$$P(j) = \begin{cases} \alpha \sum_{i \in N_j(t)} \Gamma(E_j - E_i; a, b) & \text{if } \alpha > \beta K(d_{k_j j};\ \eta) \text{ (local transmission)} \\ \beta K\left(d_{k_j j};\ \eta\right) \Gamma\left(E_{k_j} - E_i; a, b\right) & \text{otherwise (focal transmission)} \end{cases},$$

while the likelihood that j was not infected before E_j is given by $Q(E_j) = exp\ \left(-\int_0^{E_j} \lambda_j(t)dt\right)$.

The overall likelihood of a given parameter set $\theta = \{\alpha, \beta, \eta, a, b\}$ and of the ensuing transmission chain is given by:

$$L(\theta) = \prod_j P(j)\ \ Q(E_j).$$

The transmission distance $D(j)$ for each case j is defined by $d_{k_j j}$ when j was locally transmitted; for local and exported cases, we adopted the weighted average of the distances of all potential infectors, were the weights are represented by the generation time probability of each potential infector of j:

$$D(j) = \frac{\sum_{i \in C_j(E_j)} d_{ij} \Gamma(E_j - E_i)}{\sum_{i \in C_j(E_j)} \Gamma(E_j - E_i)}$$

where $C_j(t)$ corresponds to $N_j(t)$ for locally transmitted cases and to individuals infected in Anzio before time t for exported cases.

1.3. Sensitivity analysis

As a sensitivity analysis, we considered a number of alternative model versions, detailed below, where the information from epidemiological investigations was assumed to be unavailable.

1.3.1. Baseline model without epidemiological information

The model remains identical as in the main analysis, except for the fact the potential focal infector is searched among the set of all individuals in the dataset who have been infected before time t, $\mathrm{N}(t)$, independently of the location of j ($\mathrm{N}(t)$ substitutes $\mathrm{N_j}(t)$ in the expression of the force of infection). In addition, there are no cases classified as exported, therefore all cases for which a focal infector cannot be identified are considered as local, and the model is computed over all cases (n=412).

1.3.2. Power-law kernel

Here we considered a different functional form for the spatial kernel, adopting a power-law instead of a negative exponential one:

$$K(d_{ji}; \phi) = (\phi - 1)\left(\frac{1}{1+d_{ji}}\right)^{\phi},$$

with $\phi > 1$. The kernel so defined is normalized in such a way that its integral over all d_{ij} is equal to 1.

1.3.3. Demographic-specific susceptibility

In this model, we allowed parameter β to vary depending on the demographics of the infected individual, i.e. whether it was a child, c, defined as an individual of any gender with age strictly lower

than 16 years (32 cases in the dataset), an adult male, m (168 cases), or an adult female, f (212 cases). The force of infection becomes:

$$\lambda_j(t) = \sum_{i \in \mathrm{N}(t)} \left(\alpha + \beta_{\delta_j} K\left(d_{ji};\eta\right)\right) \Gamma(t - E_i; a, b)$$

where $\delta_j \in \{c, m, f\}$.

1.3.4. Seasonal transmissibility

In order to account for seasonal variations in the abundance of mosquitoes in the neighborhood of cases, and therefore on the probability of focal transmission, we assumed that transmissibility for focal infections may be dependent on time:

$$\lambda_j(t) = \sum\nolimits_{i \in \mathrm{N}(t)} \left(\alpha + \tilde{\beta}(t) K\left(d_{ji};\eta\right)\right) \Gamma(t - E_i; a, b);$$

we assumed a truncated sinusoidal function for $\tilde{\beta}(t)$:

$$\tilde{\beta}(t) = \begin{cases} \beta \ \sin\left(\dfrac{2\pi(t - t_0)}{365}\right) & for \ \ t_0 \leq \ t \leq \ t_0 + 180 \ days \\ 0 & elsewhere \end{cases}$$

With t_0 corresponding with May 15th, so that focal transmission is assumed to occur between mid-May and mid-November, with a peak probability on mid-August.

1.3.5. Step-change in transmissibility

As an alternative way to account for seasonal variations in transmission, we included a model where a step change in the probability of focal transmission is allowed [4]:

$$\tilde{\beta}(t) = \begin{cases} \beta & for \ t < \ t_0 \\ \chi\beta & for \ t \geq t_0 \end{cases}$$

Where β, χ and t_0 are free model parameters.

1.4. Calibration

The calibration for all models was performed by adopting a Monte Carlo Markov Chain (MCMC) procedure. For free model parameters (a, b, η, α, and one or more values for β, depending on model formulation), we considered recursive normal jumps and uninformative (uniform) priors. For nuisance parameters, i.e. the unknown times of infection E_j, we initialized them by subtracting from the date of symptom onset a number of days sampled from the distribution of the intrinsic incubation period [5], i.e. a uniform distribution with a minimum of 2 and a maximum of 4. At each step of the MCMC, the times of infection E_j for 10 randomly chosen individuals was updated, sampling from the same distribution. Parameter sets, together with the set of infection times E_j and the ensuing imputed transmission chain, were accepted or rejected on the basis of likelihood values according to a standard Metropolis-Hastings algorithm. We run each model for 2.5 million iterations and considered the last 500,000 MCMC samples for analysis.

Average parameter values and their 95% credible interval computed from the corresponding quantiles of the posterior distributions estimated by the MCMC procedure are reported in Table S2 for all considered models.

Table S2. *estimated parameter values for all transmission chain reconstruction models: means (in bold) and 95% credible intervals from the estimated posterior distributions.*

Parameter/ Model	Main analysis (baseline with epidemiological information)	Baseline	Power-law kernel	Demographic-specific susceptibility	Seasonal transmissibility	Step-change in transmissibility
a	**8.53**<br>7.47-9.60	**4.99**<br>4.32-5.93	**3.38**<br>3.28-3.45	**5.05**<br>4.30-6.02	**3.87**<br>3.74-4.07	**4.08**<br>3.86-4.44
b	**1.46**<br>1.21-1.77	**2.83**<br>2.08-3.59	**5.71**<br>5.52-6.09	**2.78**<br>2.03-3.61	**4.40**<br>4.01-4.67	**4.03**<br>3.43-4.46
η	**9.35 10^{-3}**<br>(8.90-9.90) 10^{-3}	**11.5 10^{-3}**<br>(6.62-14.3) 10^{-3}	-	**11.4 10^{-3}**<br>(7.20-13.9) 10^{-3}	**14.3 10^{-3}**<br>(14.2-14.5) 10^{-3}	**10.8 10^{-3}**<br>(10.6-11. 0) 10^{-3}
α	**9.45 10^{-3}**<br>(7.46-11.83) 10^{-3}	**7.59 10^{-3}**<br>(5.35-9.50) 10^{-3}	**7.76 10^{-3}**<br>(7.38-7.93) 10^{-3}	**7.75 10^{-3}**<br>(5.88-9.64) 10^{-3}	**8.26 10^{-3}**<br>(8.13-8.48) 10^{-3}	**10.2 10^{-3}**<br>(10.1-10.2) 10^{-3}
β	**35.2**<br>31.1-40.3	**32.0**<br>22.2-40.0	**46.8**<br>46.4-47.0	-	**36.1**<br>34.4-37.7	**71.3**<br>69.6-73.1
ϕ	-	-	**23.6**<br>23.3-24.3	-	-	
β_f	-	-	-	**33.5**<br>22.3-44.7	-	
β_m	-	-	-	**27.0**<br>16.7-37.7	-	
β_c	-	-	-	**60.2**<br>2.3-151.0	-	
χ						**0.199**<br>(0.122-0.275)
t_0						**Sep 18th**<br>Sep 16th – Sep 22th

To ensure convergence of the MCMC for the main analysis, we have run four parallel chains with different starting points and computed the Gelman-Rubin diagnostic R_c [6] for each parameter, finding the following values: for a: 1.45; for b: 1.43; for η: 1.23; for β: 1.11; for α: 1.18. The only diagnostics that were significantly non-compliant with the convergence criterion of Rc < 1.2 [6] are those for parameters *a* and *b*, determining the shape and scale of the Gamma-distributed generation time; this is likely due to slight variations in the accepted augmented infection times, E_j, that can result in corresponding accommodations of the curve of the generation time distribution and therefore in some correlation among the accepted parameter values. Nonetheless, posterior distributions for *a* and *b* and for the corresponding distribution of the generation time were substantially robust in the four MCMCs from the point of view of epidemiological interpretation (see Table S3).

As a further diagnostics we computed the effective sample size of the MCMC using the equation [7]

$$ESS = \frac{n}{1+2\sum_k \rho(k)}$$

where n=500,000 is the number of iterations and $\rho(k)$ is the correlation among likelihood values at lag k. We obtained a value of ESS=175.0 (ESS>100 is a generally accepted requirement for convergence of the MCMC).

Table S3. *Parameter estimates for four parallel MCMC chains in the main analysis*

MCMC chain / Parameter	*a*	*b*	Mean generation time (days)
MCMC #1	8.53 (7.47-9.60)	1.46 (1.21-1.77)	12.4 (11.7-13.3)
MCMC #2	8.15 (7.14-8.95)	1.56 (1.35-1.89)	12.6 (12.0-13.5)
MCMC #3	8.59 (7.64-9.56)	1.44 (1.22-1.72)	12.3 (11.7-13.1)
MCMC #4	8.86 (8.31-9.57)	1.37 (1.24-1.50)	12.1 (11.8-12.6)

1.5. Branching process

The branching process model attempts to reproduce the transmission of chikungunya within the municipality of Anzio using estimates for the distribution of notified secondary cases, the generation time and the spatial kernel obtained via the transmission chain reconstruction model. Each simulated outbreak is initialized with a single index case, which is marked as not notified, as in the actual outbreak. For each symptomatic case, a number *s* of symptomatic secondary cases that will be notified is first assigned by sampling from a distribution σ. In particular, we assume σ to be a Poisson distribution with rate *S*, which varies over time and is set equal to the weekly estimated average number of secondary cases in the transmission chain reconstruction model (Figure S1). Secondary cases are iteratively generated with the following characteristics:

- the date of symptom onset is assigned as $T_{new} = T_{index} + \tau$, where τ is sampled from $\Gamma(\tau; a, b)$;
- the position of the new case is assigned as $(x_{new}, y_{new}) = (x_{index}, y_{index}) + \rho \cdot (\cos(\theta), \sin(\theta))$, where ρ is sampled from the reconstructed distribution of transmission distances (Figure S2), including both local and focal cases, and θ is uniformly sampled in $[0, 2\pi)$;
- each individual may be assigned an asymptomatic status based on a Bernoulli sample with probability p_s [8];
- symptomatic individuals may be notified based on a Bernoulli sample with probability equal to a reporting rate p_d, while asymptomatic individuals will not be detected;

For each case, secondary cases are generated until the number of symptomatic notified cases exceeds *s* (the case in excess is discarded). Outbreaks were halted when they reached the same number of symptomatic notified cases as the actual outbreak, i.e. 190; outbreaks resulting in stochastic extinctions in the first weeks were discarded and re-simulated.

All parameters and probability distributions for the branching process are either obtained from literature or estimated from the transmission chain reconstruction model (Table S4). We assume a baseline value for the reporting rate of 100% in the main analysis and show the model behavior when its value is reduced to up to 10% of all incident cases. In addition, we show the model performance when using different values of the notification rate for symptomatic cases before and after outbreak discovery on September 7. To do so, we switch the value of the notification rate when the number of notified symptomatic cases in the branching process reaches the value of 93, i.e. the number recorded in the Anzio outbreak on September 7. Results are reported in section 3.

Table S4. *Parameters for the branching process*

Parameter	Sampling	Distribution parameters	Source
Number of secondary symptomatic notified cases, S	Poisson	Time-varying (Fig. S1)	Transmission chain reconstruction model
Generation time, τ	Gamma	Shape: 8.53<br>Scale: 1.46	Transmission chain reconstruction model
Transmission distance, ρ	Empirical (Fig. S2)	-	Transmission chain reconstruction model
Transmission direction, θ	Uniform	Min: 0<br>Max: 2π	Assumption
Symptomatic cases	Bernoulli	p_s = 81.8%	[8]
Baseline reporting rate for symptomatic cases	-	p_D = 100%	Assumption; sensitivity analysis over range 10-100%.

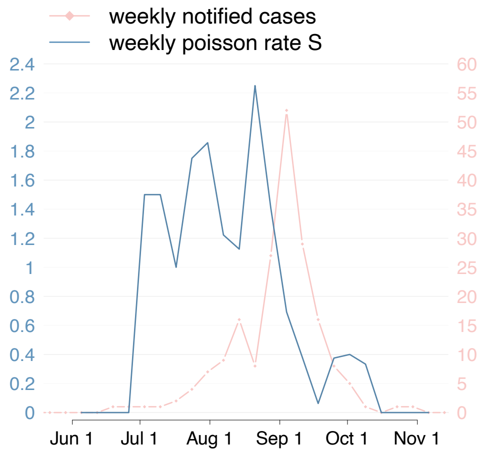

Figure S1. *Poisson rates over time used for sampling the number of secondary symptomatic notified cases.*

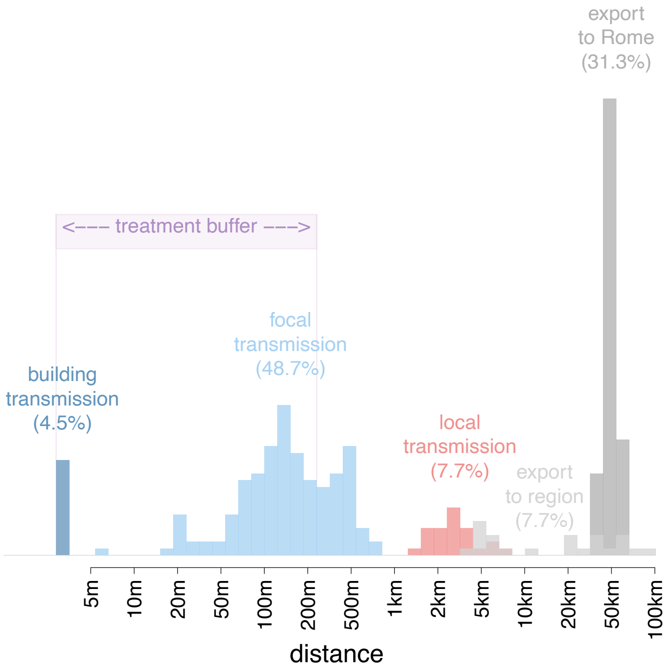

Figure S2. *Overall distribution of the estimated transmission distances by mode of transmission, considering only cases transmitted within Anzio, i.e. excluding exportations*.

1.6. Spatial mechanistic model

We developed a spatial mechanistic model of chikungunya transmission and insecticide spraying interventions to evaluate, on one hand, the effect of different intervention protocols, and on the other hand, the effect of long-distance mobility on the overall effectiveness of the currently adopted protocol.

The model represents a spatial square of side approximately 20km, divided into a 251x251 grid of cells of size l. Each cell was initialized with a total number of human inhabitants and mosquitoes sampled from a Poisson distribution. For humans, the Poisson rate was given by $n_h = D_h l^2$, where D_h is the average population density of the residential area of Anzio. Similarly, for mosquitoes we consider a Poisson rate given by $n_m = D_m l^2$, where the density of mosquitoes D_m is computed by inverting the classical equation for R_0 [9]:

$$D_m = R_0 D_h \gamma \mu \frac{\mu + \omega}{k^2 \chi_m \chi_h \omega}$$

We chose R_0 as the average number of secondary transmissions in absence of interventions, as estimated by the transmission chain reconstruction model; all other epidemiological parameters (see Table S5) were taken from the literature. The same parameters were used in the model to determine epidemiological transitions within a classical SEIR-SEI structure. A single infectious individual is introduced on June 25th (day of symptom onset for the first case detected in Anzio) in the central cell of the grid. We assumed that mosquitoes are at population equilibrium, so that in each cell and at each time step (1/1000th of a day), new susceptible adult mosquitoes replace dying mosquitoes. Mosquitoes can acquire the virus by infectious individuals residing within a square of side 5 pixels (corresponding to a radius of action of about 200m, which is approximately the flight range of *Aedes albopictus* [10]). Thus, at each time step, mosquitoes in a given cell with coordinates *i, j*, are subject to a force of infection given by

$$\lambda_m[i,j] = k\chi_m \frac{\sum_{X=i-2}^{i+2}\sum_{Y=j-2}^{j+2} I_h[X,Y]}{\sum_{X=i-2}^{i+2}\sum_{Y=j-2}^{j+2} N_h[X,Y]}$$

where I_h and N_h are the respectively the number of infectious and total individuals in a given cell.

Similarly, we assumed that human infections produced by mosquitoes belonging to a given cell depend on the number of humans residing in neighboring cells with a force of infection

$$\lambda_h[i,j] = k\chi_h \frac{I_m[i,j]}{\sum_{X=i-2}^{i+2}\sum_{Y=j-2}^{j+2} N_h[X,Y]};$$

To account for human mobility, the number of resulting new infections is distributed over the grid according to the estimated distribution of transmission distances in Anzio for focal and local cases (Figure S2), choosing at random the direction of displacement.

Insecticide spraying interventions are modeled as follows: after outbreak detection (occurring at time T in the simulation), any new infectious case triggered treatment in a square of size 2ρ centered on the residence of the new case, with a delay after symptom onset that was uniformly sampled over the interval [1, 2τ] days. Treatment resulted in the sudden death without replacement of a proportion ϕ of all susceptible, exposed and infectious mosquitoes in treated cells; the mosquito population recovered to pre-treatment equilibrium population sizes after a fixed number of days Θ (Table S5). T, ρ and τ are considered parameters of the intervention protocols and are changed one at a time in the different scenarios shown in the main analysis (Figure 6).

Table S5. *Parameters for the spatial mechanistic model*

Parameter	Value	Unit	Source
Size of cell, l	80	m	-
Human population density, D_h	30.0	Individuals/ha	[1]
Duration of infectious period in humans, $1/\gamma$	4.5	days	[9]
Average number of secondary cases, R_0	1.4	-	Transmission chain reconstruction model
Mosquito life span, $1/\mu$	6.8	days	[9]
Duration of incubation period in mosquitos, $1/\omega$	2.5	days	[9]
Duration of incubation period in humans, $1/\omega_h$	3	days	[5]
Mosquito biting rate, k	0.09	bites/mosquito/day	[11]
Probability of human to mosquito transmission per bite, χ_m	77	%	[9]
Probability of mosquito to human transmission per bite, χ_h	70	%	[9]
Effectiveness of insecticide spraying in reducing the mosquito population, ϕ	86	%	[12]
Time to recovery of the mosquito population, Θ	14	days	[12]

2. Sensitivity analyses with respect to different model formulations

2.1. Baseline model

Considering a random subsample of 10,000 reconstructed chains in the baseline model (out of a total of 500,000) rather than only the most likely presented in the main text, we found a very limited variability in the identified infectors. In particular, 60% of all non-exported cases were consistently assigned the same infector in all transmission chains; in addition, a further 36% of them were assigned the same infector in more than three quarters of transmission chains; only for the remaining 4% the candidate infector was more uncertainly identified. The proportion of focally acquired cases varied from 54.8% to 55.1%. Therefore, the conclusions of this study are robust with respect to variability in reconstructed transmission chains.

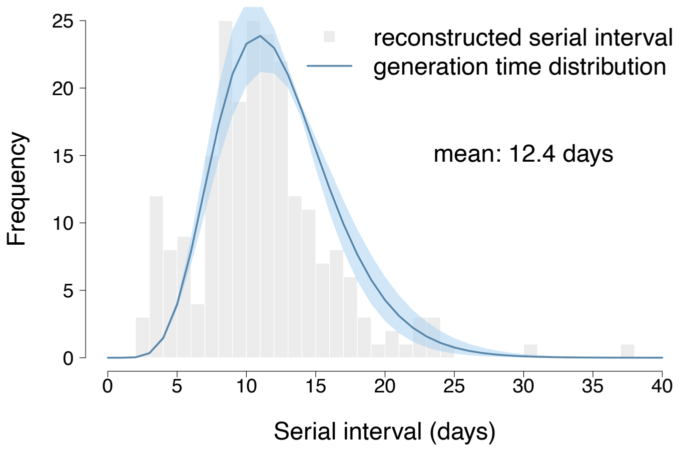

Figure S3. Reconstructed serial interval and probability distribution function of the generation time.

Table S6. *Quantitative estimates for all transmission chain reconstruction models. We report the central value of the distribution (in bold) and the 95% credible interval. *: central value is the mean; #: central value is the median.*

Output variable	Main analysis (baseline with epidemiological information)	Baseline	Power-law kernel	Demographic-specific susceptibility	Seasonal transmissibility	Step-change in transmissibility
Generation time (days)*	**12.4**<br>11.7-13.3	**14.0**<br>12.4-15.5	**16.5**<br>14.3-20.3	**13.9**<br>12.2-15.5	**15.3**<br>13.7-17.8	**16.4**<br>15.2-17.2
Transmission distance (m) *	**174**<br>169-179	**154**<br>119-251	**94**<br>51-114	**150**<br>117-230	**116**<br>102-128	**199.5**<br>187.8-213.7
Number of clusters of size >3 #	**11**<br>10-11	**18**<br>15-22	**15**<br>13-18	**18**<br>15-22	**15**<br>13-18	**18**<br>16-21
Number of clusters of size > 10 #	**3**<br>3-3	**4**<br>3-5	**4**<br>2-5	**4**<br>3-5	**5**<br>3-5	**4**<br>3-6
Max cluster duration (days) #	**132**<br>132-132	**108**<br>92-108	**95**<br>88-108	**108**<br>92-108	**108**<br>92-108	**108**<br>108-108
Cluster size *	**25.6**<br>24.6-26.8	**11.0**<br>9.7-13.3	**10.4**<br>7.1-12.1	**10.7**<br>9.4-12.6	**11.2**<br>9.8-12.7	**13.5**<br>12.1-15.2
% of cases classified as focal #	**54.9**<br>54.8-55.1	**58.7**<br>53.1-67.7	**50.6**<br>37.7-53.1	**57.7**<br>52.3-63.8	**53.1**<br>51.3-54.3	**66.7**<br>66.2-66.9
% of cases within 200m #	**38.3**<br>37.6-39.1	**41.3**<br>35.4-44.0	**44.8**<br>37.4-46.7	**41.1**<br>36.0-43.8	**43.6**<br>41.3-45.7	**40.5**<br>38.1-42.4

Figure S3 shows the histogram of serial intervals for reconstructed focal transmissions (maximum likelihood estimate) and the corresponding probability distribution function of the generation time estimated by the MCMC procedure (average and 95%CI computed from the joint posterior distribution of *a* and *b*).

In Table S6, we report a comparison of the main quantitative estimates across the different models and reconstructed transmission chain. In the remainder of this section we show results computed using the maximum-likelihood transmission chains for each model.

2.2. Baseline model without epidemiological information

The model still identifies two main clusters in Anzio, A1 and A2, with 78 and 57 focally transmitted cases overall, and one in Rome (with 20 secondary cases); the average speed of diffusion remained in the same range (300-600 m/month; Figure S4). The transmission distance was slightly longer compared to the main analysis, with a median of about 200m (instead of 127m), and this resulted in a slightly larger proportion of cases (68.9%, compared to 54.9% in the main analysis) being classified as focal (Figure S5). This increase in the proportion of focal cases also resulted from the identification of plausible focal infectors for cases which were exported from Anzio. The distribution of distances for local transmission in this model shows a clear bimodality, with a first peak between 1 and 20km and a second peak, substituting cases which were classified as exported in the main analysis, between 20 and 70km.

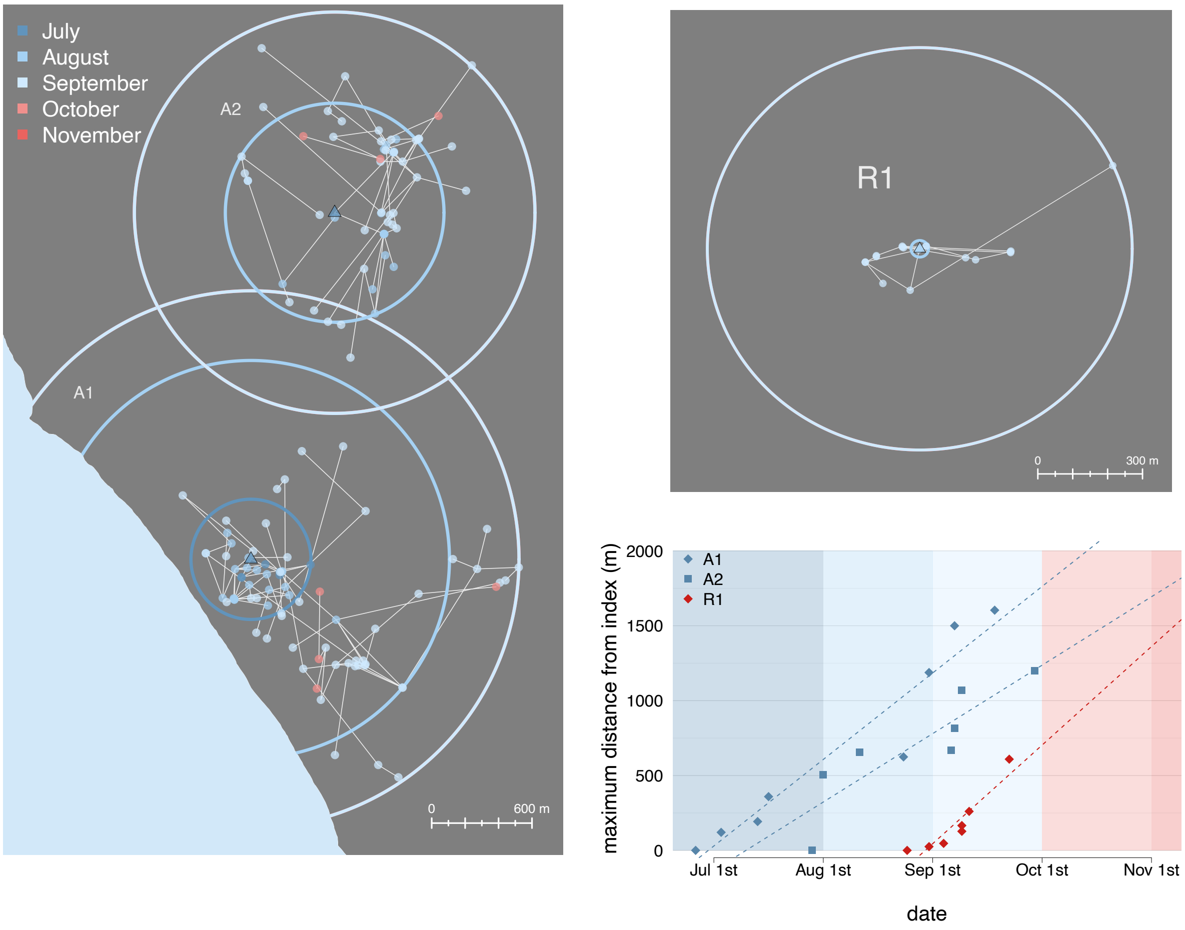

Figure S4. Baseline model with no epidemiological information: main clusters and clusters spread.

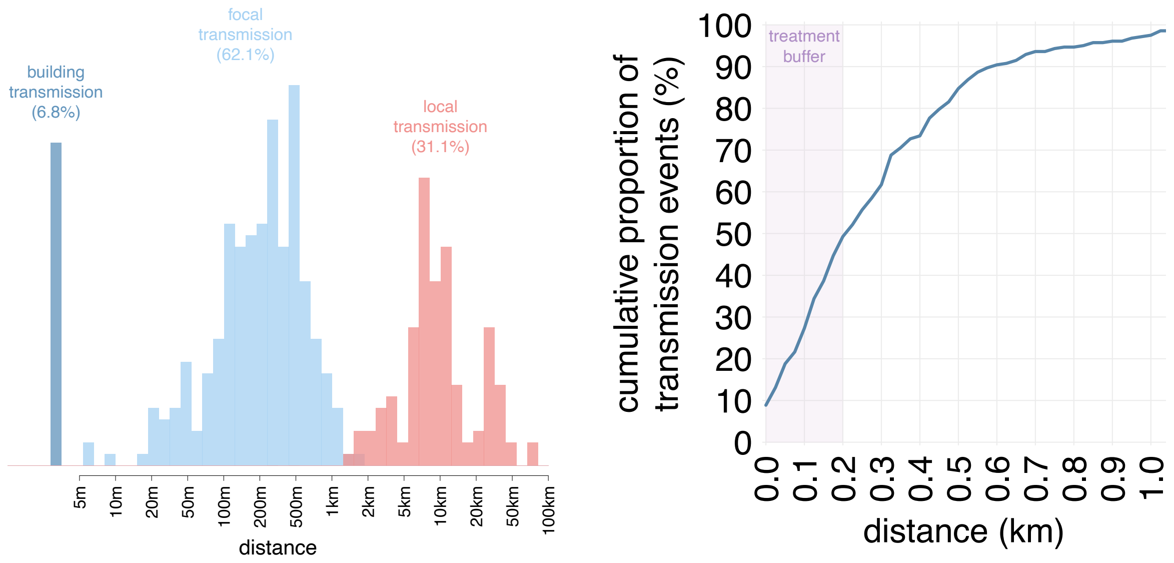

Figure S5. Baseline model with no epidemiological information: transmission modes and cumulative distribution of focal transmission distance

2.3. Power-law kernel

The model with a power-law kernel does not deviate significantly from the negative-exponential kernel shown in the main analysis: parameters estimated in the two cases lead to practically identical probability distribution functions (see Figure S6). All results from the ensuing reconstruction of the transmission chains are also quite consistent with the main analyses, except for the fact that cluster A2 is split into two separate clusters (A2 and A3, Figure S7). The speed of the cluster diffusion is, again, between 250 and 400 m/month. The proportion of focally transmitted cases is estimated at about 59% and, of these, about 75% occur within 200m (Figure S8).

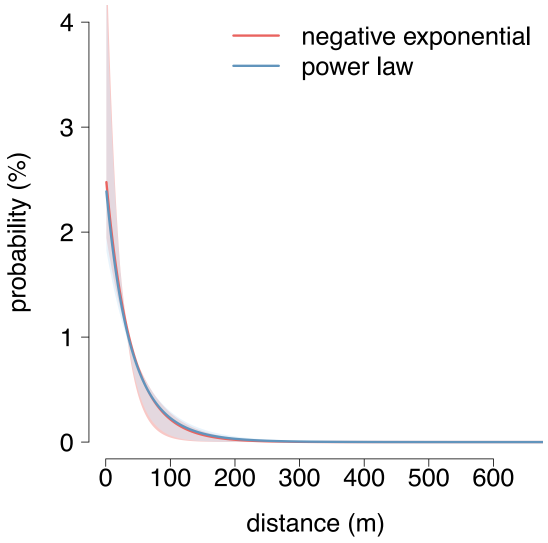

Figure S6. Comparison between estimated negative exponential and power law kernels.

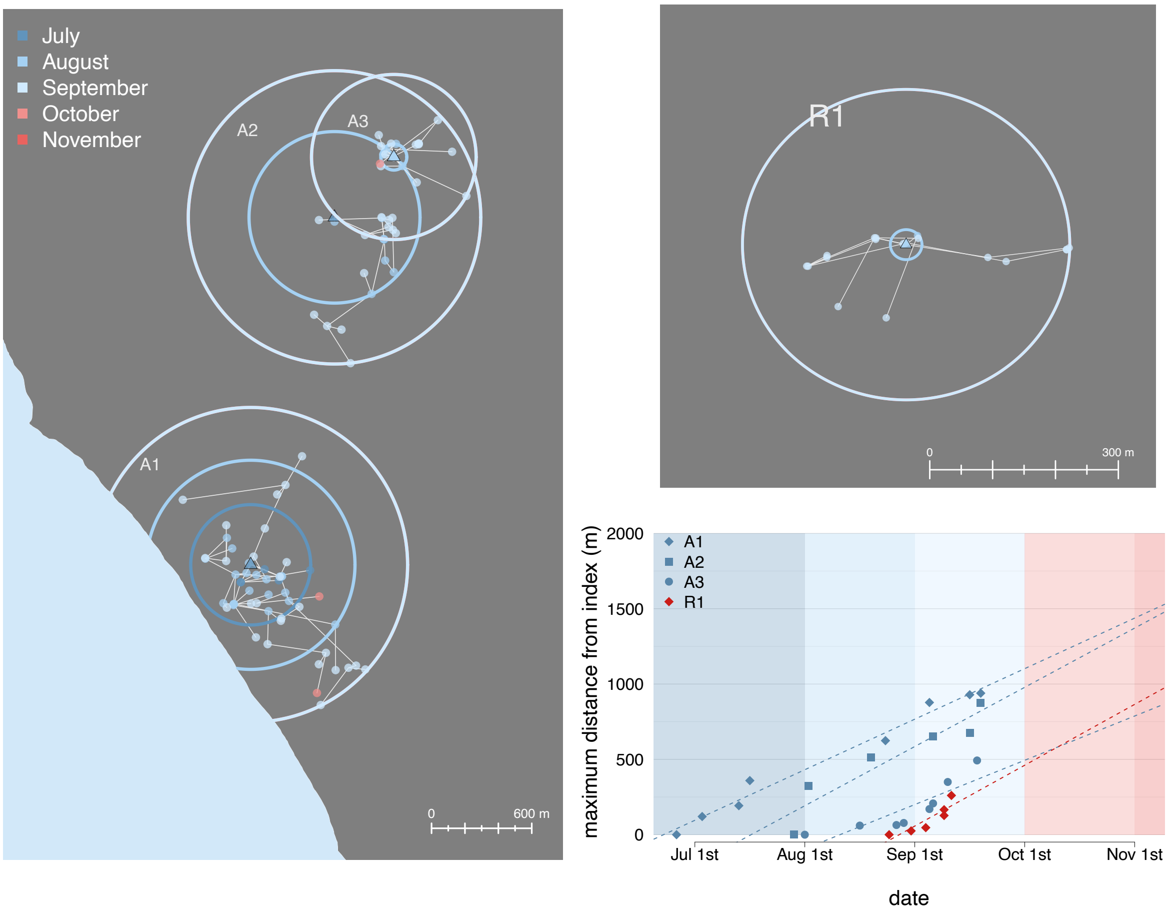

Figure S7. Power-law kernel model: main clusters and clusters spread.

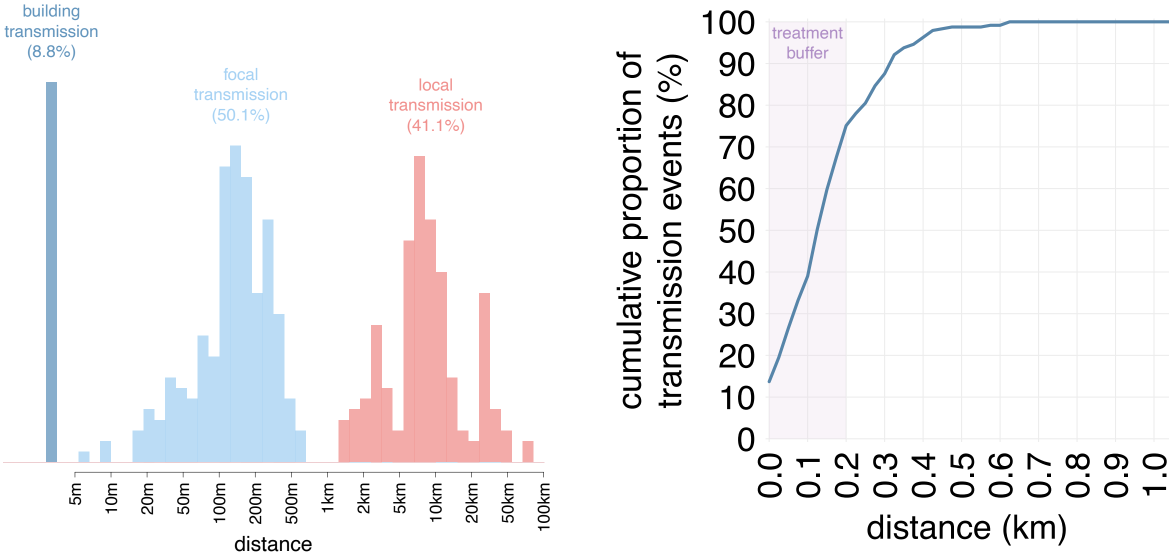

Figure S8. Power-law kernel model: transmission modes and cumulative distribution of focal transmission distance.

2.4. Demographic-specific susceptibility

Conclusions from the main analysis are also robust when considering a differential susceptibility by demographic class (adult males and females, children). The main clusters and the relative size were still the same (Figure S9) with a diffusion speed of 350-650m/month; the proportion of focal cases (68.1%) and the transmission distances (median: 175m) were similar to the model with uniform susceptibility across demographic classes shown in Section 2.2 (Figure S10). Significant differences in the estimated values of susceptibility for different classes (Table S2) did not result in a higher proportion of transmitted cases by any demographic class, just as in the main analysis (Figure S11).

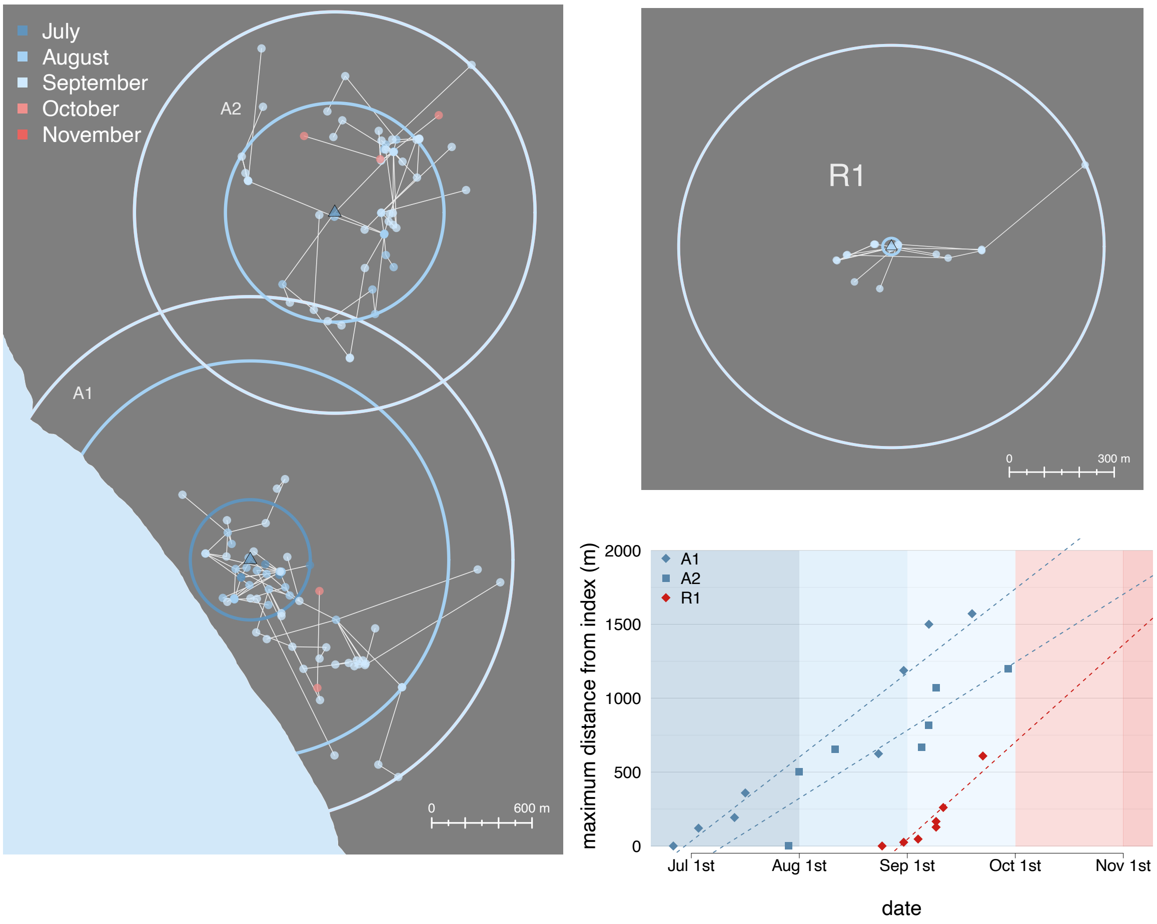

Figure S9. Model with demographic-specific susceptibility: main clusters and clusters spread.

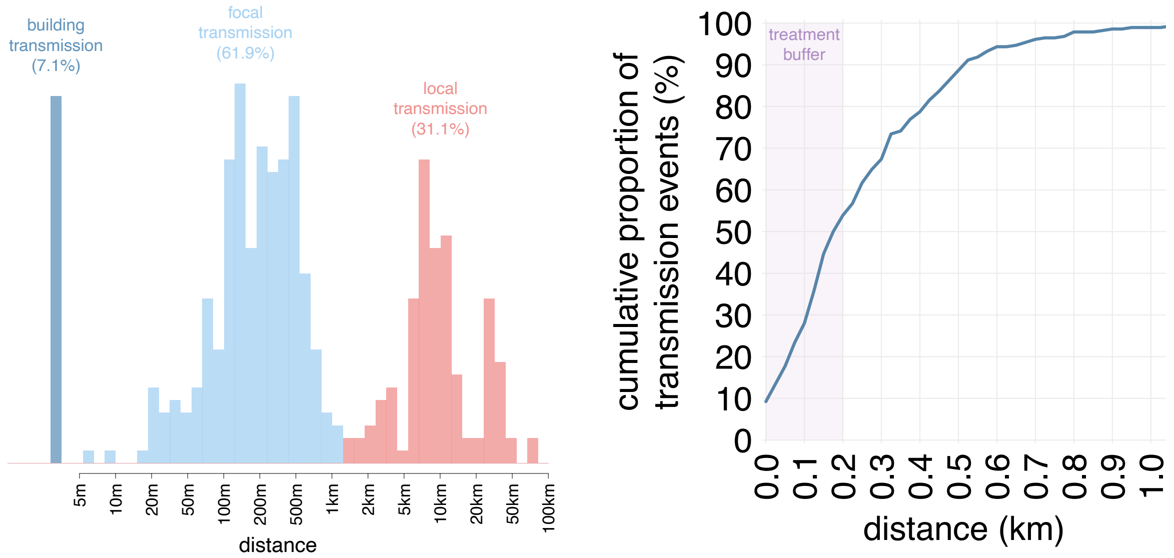

Figure S10. Model with demographic-specific susceptibility: transmission modes and cumulative distribution of focal transmission distance.

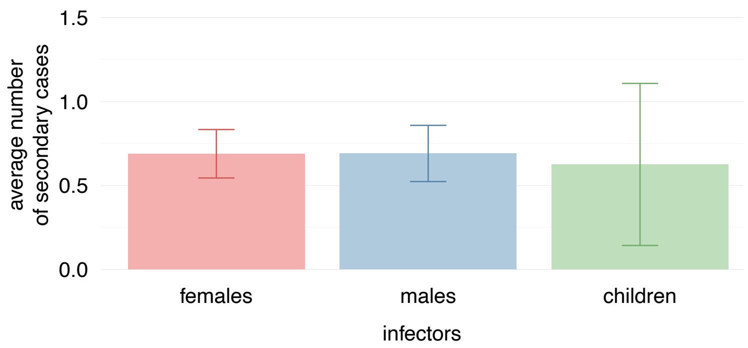

Figure S11. Model with demographic-specific susceptibility: average number of secondary cases by demographics

2.5. Seasonal transmissibility

Results from the model with seasonal transmissibility were also substantially similar to all other analysis. Cluster A2 is split into two simultaneous and close clusters because of the reduced transmissibility in the early part of the outbreak; diffusion speeds were 190-450 m/month (Figure S12). 62.6% of cases were classified as focal and 70% of these were transmitted within 200m (Figure S13).

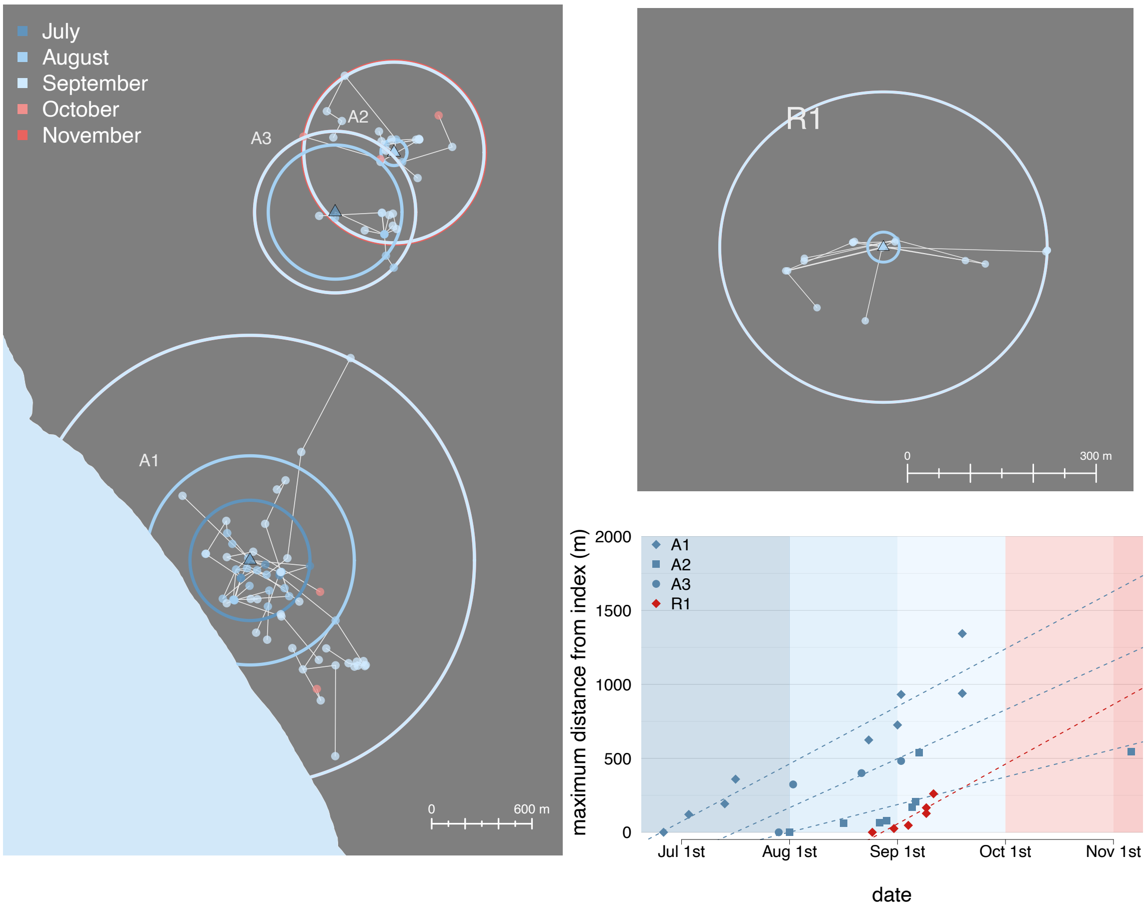

Figure S12. Model with seasonal transmissibility: main clusters and clusters spread.

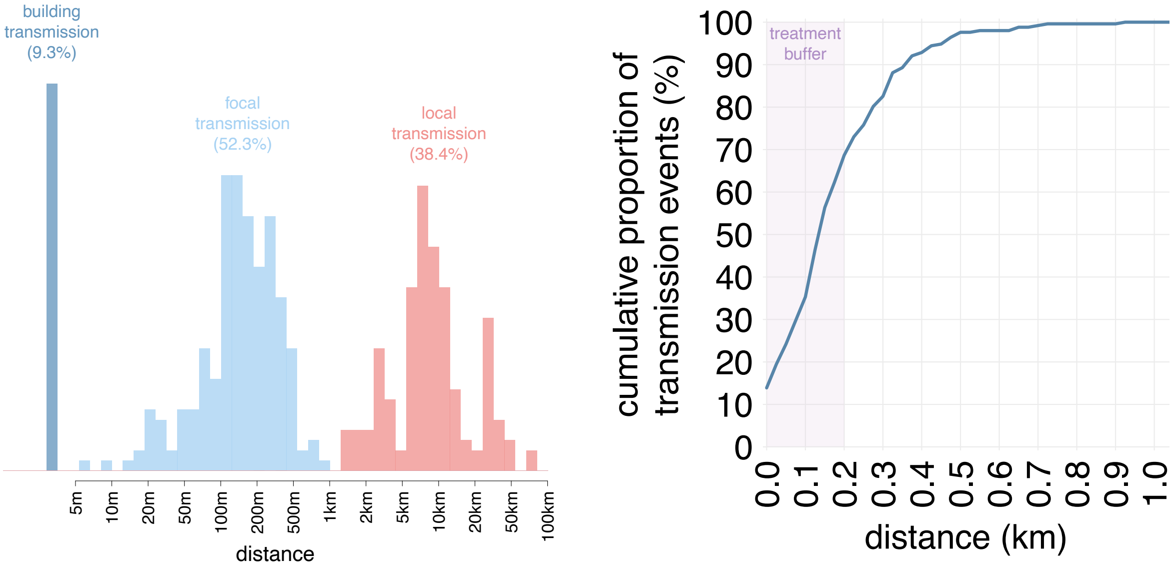

Figure S13. Model with seasonal transmissibility: transmission modes and cumulative distribution of focal transmission distance.

2.6. Model with step-change in transmissibility

When considering the possibility of a step-change in transmissibility, the model estimated an 80.1% (95%CI 72.5-87.8%) relative reduction in transmission around September 18th (95%CI Sep 16th – Sep 22nd), i.e. just a few days after systematic treatment in the area had been implemented on September 14th. It is tempting to consider this result as an estimate of treatment effectiveness; however such an interpretation would be far-fetched, since mosquito population reductions due to falling temperatures in the same period of the year may have also played a significant role. In any case, the reconstructed spatiotemporal dynamics were analogous to those identified in the main analysis: the diffusion speed of the three main clusters were 180-600m/month (Figure S14), 67.2% of transmissions were classified as focal and 60% of them were transmitted within 200m (Figure S15).

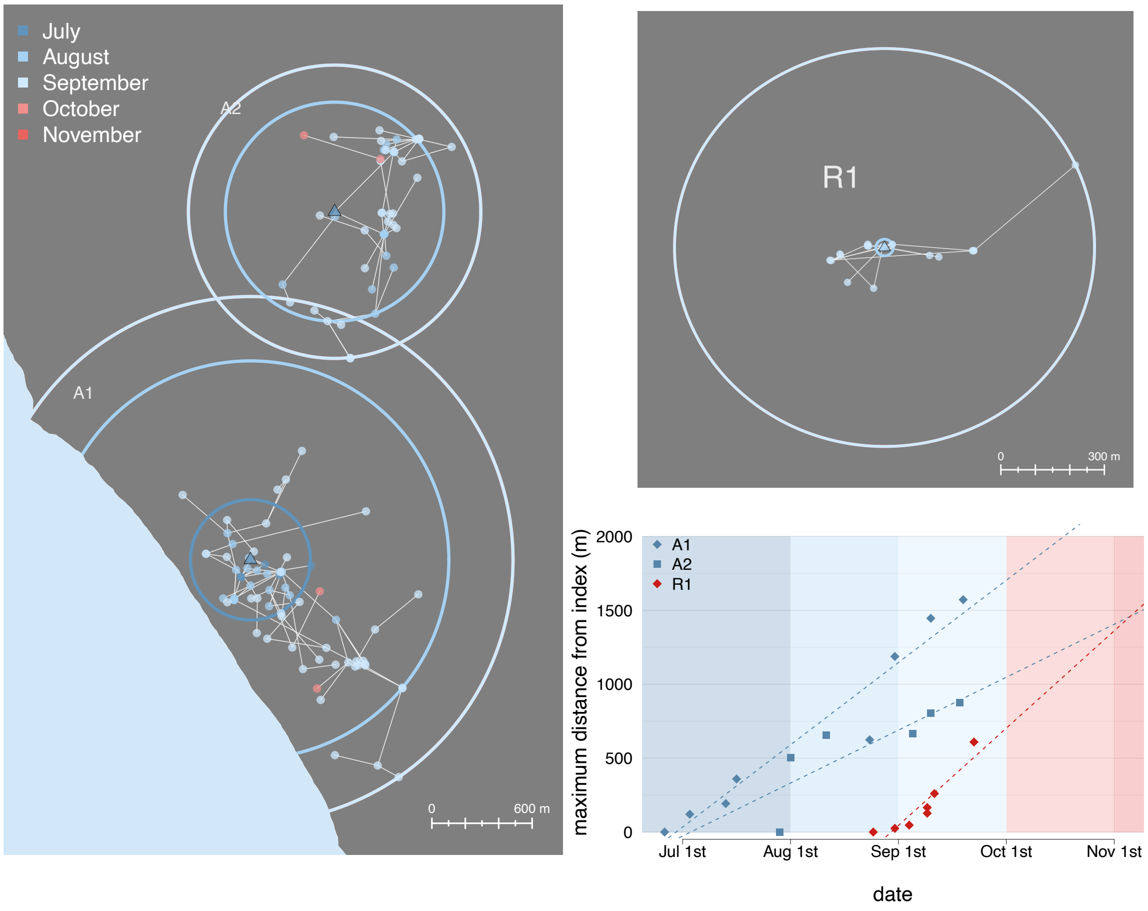

Figure S14. Model with step change in transmissibility: main clusters and clusters spread.

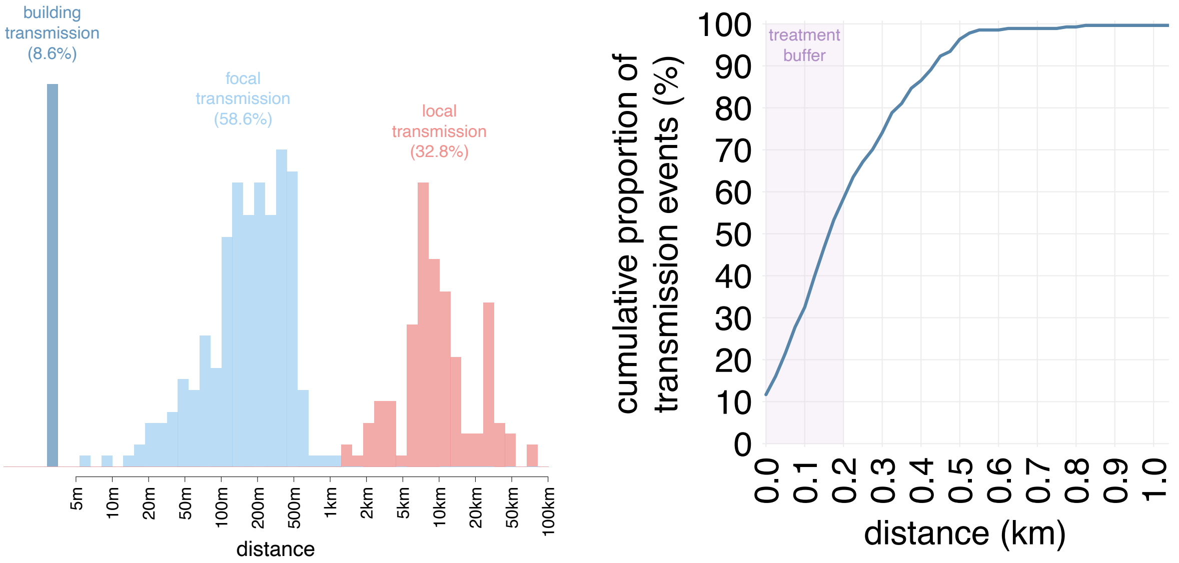

Figure S15 Model with step change in transmissibility: transmission modes and cumulative distribution of focal transmission distance.

3. Robustness of branching process results

Figure S16 shows that the final outbreak diameter was substantially unaffected by the assumption on the value of the reporting rate, and that reporting rates p_D below 50% are implausible: in such case, the outbreak would be expected to evolve over a much shorter time scale compared to observations, due to widespread background transmission.

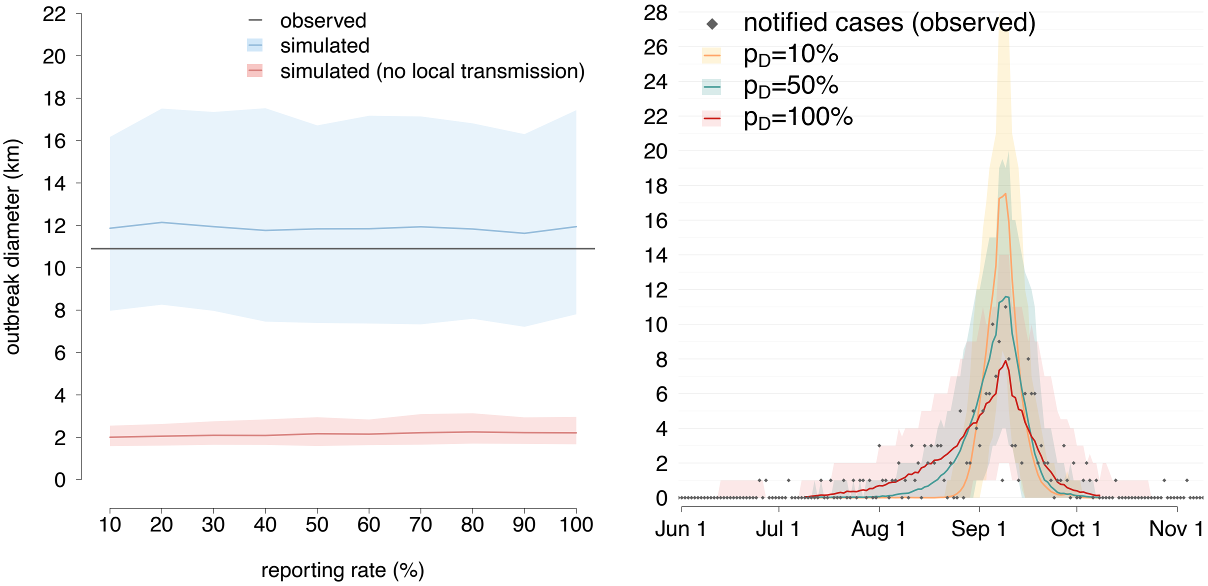

Figure S16. Effect of the reporting rate p_D on the final diameter and temporal evolution of simulated outbreaks, compared with corresponding observations.

Figure S17 shows the mean squared error between the observed number of notified symptomatic cases per week and corresponding model predictions, using different combinations of the notification rate before and after outbreak detection on September 7. The figure confirms that the best model performance occurs when the notification rate of symptomatic cases is above 70% before outbreak detection and between 90 and 100% after outbreak detection. For reference, the 2007 outbreak in Emilia Romagna, which was characterized by similar timing, intervention delay and surveillance effort [13], had an overall 78% reporting rate of symptomatic cases [8].

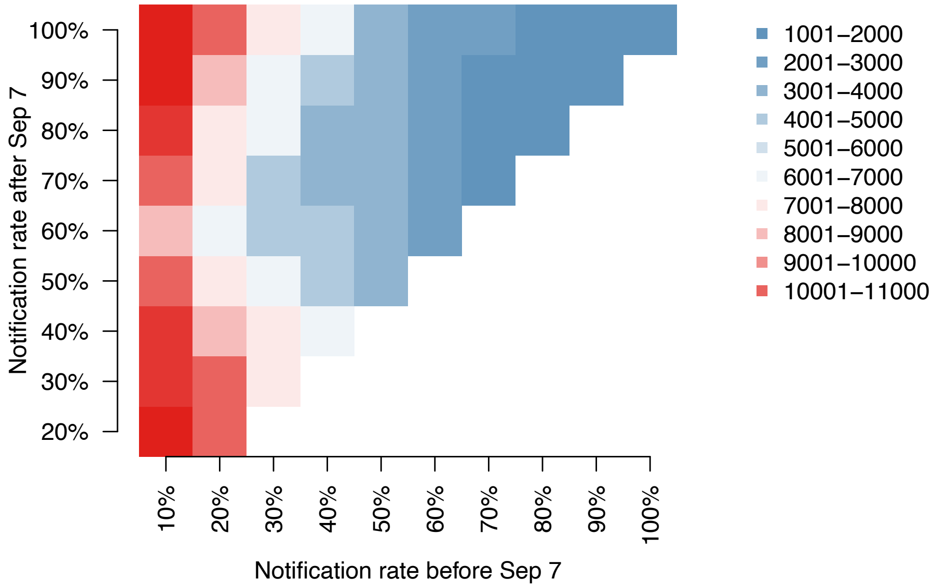

Figure S17. *Mean squared error between model observed weekly number of symptomatic notified cases and corresponding model estimates using the branching process with different values of the notification rates before and after outbreak detection (September 7).*

4. Impact of long-distance transmission on the effectiveness of interventions

Here, we show additional results obtained with the spatial mechanistic model under the baseline protocol of intervention (ρ=200m, τ=6 days and T=7th September) and two alternative scenarios for human mobility: one where local transmission is not allowed ("focal" scenario), and one where only focal transmission within 200m is considered ("hyperfocal" scenario). Results show that about 47% (95%CI: 36-58%) and 81% (95%CI: 71-91%) less cases after outbreak detection would be expected in the focal and hyperfocal scenarios respectively (Figure S18). This outcome depends on the fact that secondary cases residing within a buffer of 200m from their infector will have a much lower probability of onward transmission, because the treatment performed for their infector has lowered significantly the mosquito abundance in the surrounding of their own residence: thus, the higher the proportion of cases transmitted at short distances, the better the improvement in control. This results also shows that, while current protocols for outbreak control are likely appropriate in territories with low human mobility [2], the frequent spread of infection over long distances observed for the Lazio outbreak may have significantly hurdled the ability of adopted interventions to effectively curb transmission.

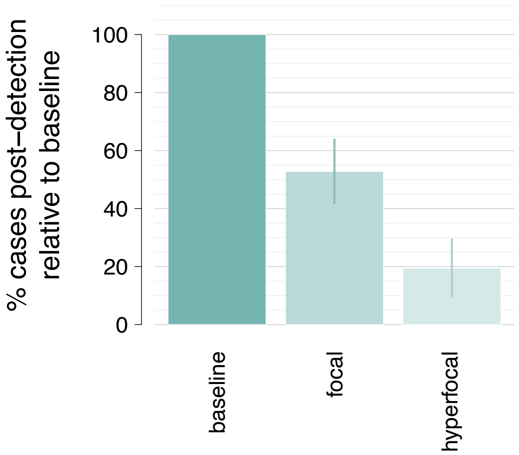

Figure S18. Effect of the alternative human mobility scenarios on the potential effectiveness of the current protocol of insecticide spraying for infection control.

5. Additional results

5.1. Statistics on average numbers of secondary cases

In Figure 4 of the main analysis we show the number of secondary cases focally transmitted by each individual, disaggregated by different covariates, i.e. demographics (adult females and males more than 15 years old, children of age below or equal 15 years), age, and population density in a 500m radius of the case. To assess the statistical significance of these relationship, we computed Generalized Linear Models assuming a Poisson distribution of the independent variable (the number of secondary cases) with respect to the explanatory variable. For the demographic disaggregation, we found non-significant relative coefficients for either adult males (p=value: 0.164) or children (p-value: 0.340) with respect to adult females, supporting the conclusion of no difference in transmission among groups. Similarly, we found a p-value of 0.72 when age was considered as a predictor of the number of transmitted secondary cases. Conversely, the population density had a significant effect on transmission, with a p-value of $6.8\ 10^{-7}$.

Bibliography

1. https://ghsl.jrc.ec.europa.eu/download.php; accessed on Oct 24, 2019
2. https://www4.istat.it/it/archivio/209722; accessed on Oct 24, 2019
3. Guzzetta G, Marques-Toledo CA, Rosà R, Teixeira M, Merler S. Quantifying the spatial spread of dengue in a non-endemic Brazilian metropolis via transmission chain reconstruction. Nat Comm. 2018;9:2837.
4. Salje H, Lessler J, Paul KK, Azman AS, Rahman MW, Rahman Met al. How social structures, space, and behaviors shape the spread of infectious diseases using chikungunya as a case study. Proc Natl Acad Sci. 2016;113:13420-5.
5. Thiberville SD, Moyen N, Dupuis-Maguiraga L, et al. Chikungunya fever: epidemiology, clinical syndrome, pathogenesis and therapy. Antiviral Res. 2013;99(3):345-70. https://doi.org/10.1016/j.antiviral.2013.06.009 PMID: 23811281
6. Brooks S, Gelman A. Some issues for monitoring convergence of iterative simulations. Computing Science and Statistics. 1998:30-6.
7. Ripley BD. Stochastic simulation. John Wiley & Sons.
8. Moro ML, Gagliotti C, Silvi G, et al. Chikungunya virus in North–Eastern Italy: a seroprevalence survey. Am J Trop Med Hyg. 2010; 82: 508–511.
9. Poletti P, Messeri G, Ajelli M, Vallorani R, Rizzo C, Merler S. Transmission potential of chikungunya virus and control measures: the case of Italy. PLoS One. 2011 May 3;6(5):e18860.
10. Marini M, Caputo B, Pombi M, Travaglio M, Montarsi F, Drago A, et al. Estimating spatio-temporal dynamics of *Aedes albopictus* dispersal to guide control interventions in case of exotic arboviruses in temperate regions. Sci Rep. 2019;9:10281.
11. Guzzetta G, Poletti P, Montarsi F, Baldacchino F, Capelli G, Rizzoli A, Rosà R, Merler S. Assessing the potential risk of Zika virus epidemics in temperate areas with established Aedes albopictus populations. Eurosurveillance. 2016 Apr 14;21(15):30199.
12. Manica M, Cobre P, Rosà R, Caputo B. Not in my backyard: effectiveness of outdoor residual spraying from hand-held sprayers against the mosquito Aedes albopictus in Rome, Italy. Pest management science. 2017 Jan;73(1):138-45.
13. Caputo B, Russo G, Manica M, Vairo F, Poletti P, Guzzetta G, et al. A comparative analysis of 2007-2017 Italian Chikungunya outbreaks and implication for public health response. Plos Negl Trop Dis, 2020. In press.
